# Lung cell fates during influenza

**DOI:** 10.1038/s41422-025-01163-y

**Published:** 2025-08-18

**Authors:** Brianna Jarboe, Maria Shubina, Ryan A. Langlois, David F. Boyd, Siddharth Balachandran

**Affiliations:** 1 Drexel University College of Medicine, Philadelphia, PA USA; 2 Center for Immunology, Fox Chase Cancer Center, Philadelphia, PA USA; 3 Department of Microbiology and Immunology, University of Minnesota, Minneapolis, MN USA; 4 Department of Molecular, Cell, and Developmental Biology, University of California, Santa Cruz, CA USA

**Keywords:** RIG-I-like receptors, Autoimmunity

## Abstract

Roughly 1 billion people are infected by Influenza A viruses (IAVs) worldwide each year, resulting in approximately half a million deaths. Particularly concerning is the threat of IAV spillover from avian and other animal reservoirs. The recent outbreak of highly pathogenic avian influenza H5N1 in US dairy cows highlights this concern. While viruses that enter human populations from such zoonotic transmission typically lack the ability to transmit effectively between humans, they may be only a few mutations from acquiring this capacity. These newly adapted viruses have the potential to be significantly more virulent than seasonal strains. A major contributor to influenza pathology is the over-exuberant immune response to the virus, particularly when the infection is present in distal pulmonary tissues. Maladaptive immune pathway over-activation can drive tissue damage and pathology, often independently of effective viral control. Anti-inflammatories targeting host-initiated pathological processes hold promise, but these avenues require a thorough understanding of virus-triggered lung inflammation before they can be fully exploited. In this review, we will discuss recent advances in our understanding of the cell types that are targeted by IAV, the consequences of IAV infection on the biology of these cells, and their contribution to lung pathology in influenza. We will also discuss how virus-induced hyper-inflammatory responses present new entry-points for therapeutic intervention, showcasing Z-form nucleic acid-binding protein 1 (ZBP1)-initiated necroptosis as an example of one such pathway.

## Introduction

There are four known types of influenza viruses, Influenza A, B, C and D, all belonging to *Orthomyxoviridae*, a family of enveloped viruses with negative sense single-stranded RNA genomes. In humans, influenza viruses cause infection of the upper and lower airways, with infection of the lower respiratory tract correlating with more severe lung pathology and disease.^1^ Influenza viruses are readily spread by aerosol and by contact with contaminated surfaces.^2^ While all four types of influenza viruses are known to infect humans, Influenza D virus infections are largely asymptomatic, and Influenza C virus infections are typically very mild and mainly restricted to children.^3^ Influenza A virus (IAV) and Influenza B virus infections, on the other hand, are the dominant causes of influenza in humans and are responsible for seasonal epidemics.^4^ Additionally, IAV infections are thought to cause the majority of cases requiring hospitalization or leading to mortality.^5,6^ IAV strains have also been the cause of several major pandemics in recent memory including the most lethal yet, the 1918 pandemic, as well as the recent 2009 A (H1N1), ‘Swine Flu’ pandemic.^5,6^ Consequently, IAV has received the most pre-clinical and clinical attention and will be the primary focus of this review.

IAV infects several vertebrate groups, including wild birds, domestic poultry and swine, dogs, horses, and bats.^7,8^ Wild aquatic birds are considered the primary natural reservoir of IAV diversity, although bats, particularly those from the New World, have been found to display a remarkable diversity of IAV subtypes.^9^ Transmission of avian IAV from wild birds to livestock and other mammals occurs by varied routes of transmission, such as through the oral-fecal route and via shared water sources.^7^ IAV strains had previously demonstrated limited infection efficiency and transmission in cattle, making the recent highly pathogenic avian influenza (HPAI) H5N1 outbreak in dairy cows unprecedented.^10,11^ Mammary tissue appears to be the primary site of viral replication in H5N1-infected cows, with high viral loads detected in their milk, suggesting that viral contamination of milk and milking machinery may be a major route of transmission between cows, and potentially to farm workers.^10^ While H5N1 seems to be efficiently spread between cattle, transmission to humans has remained limited and there has yet to be any documented human-to-human transmission.^10^

The presence of either an α-2,3 or an α-2,6 glycosidic bond at the secondary terminal galactose of the sialic acid residues, displayed on the extracellular surface of proteins, has historically been considered a major determinant of virus tropism.^12,13^ Avian strains of IAV show strong bias towards infecting cells with α-2,3-linked sialic acids, while human-adapted strains exhibit a preference for cells with α-2,6-linked residues.^14^ This predilection is partially explained by the observation that α-2,6-linked sialic acids predominate on cells of the human upper respiratory tract, where infection is initiated.^14^ Bovine mammary epithelial cells, which appear to serve as the primary replication site for the H5N1 virus in the recent cattle outbreak, intriguingly express both α-2,3-linked and α-2,6-linked sialic acid receptors.^10^ Additionally, in the limited cases of HPAI H5N1 in humans, infections have manifested primarily with mild respiratory symptoms and conjunctivitis.^10^ Notably, human conjunctival cells are also known to express α-2,3-linked sialic acids.^15^

**Fig. 1:**
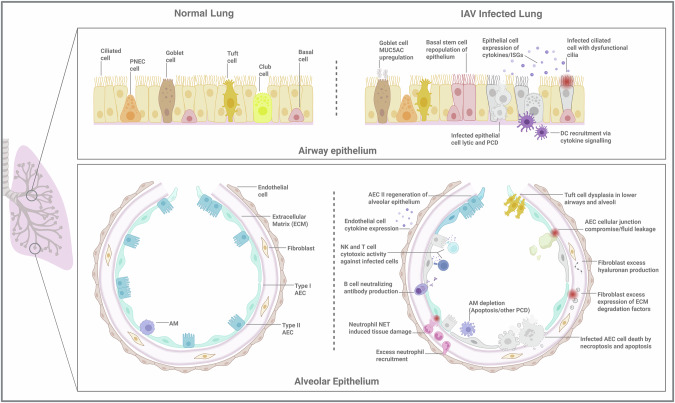
Features of IAV infection in lung cell types of the airway and alveolar epithelia. The airway epithelium (top) is composed of ciliated, goblet, club, basal, tuft cells and PNECs, which together enable MCC and maintain airway barrier integrity during homeostasis. IAV-infected epithelial cells undergo widespread lytic and programmed cell death. Even in the absence of cell death, infection can disrupt key cellular functions, such as ciliary activity. Protective host responses include increased secretion of mucin MUC5AC, and basal cell-driven epithelial regeneration. ISGs and cytokines, expressed by both infected and bystander epithelial cells, amplify antiviral responses and promote the recruitment of immune cells, such as DCs, to sites of infection. The alveolar epithelium **(**bottom**)** is the parenchymal tissue of the lung and is primarily composed of type I and type II AECs. Other significant cell types making up alveoli include endothelial cells, fibroblasts, and AMs. During infection, endothelial cells are significant producers of cytokines promoting immune cell recruitment, including that of cytotoxic T and NK cells, which can restrict infection, but also cause epithelial injury depending on disease severity. AM depletion hampers antiviral defense while excessive neutrophil recruitment and NET formation cause tissue damage. Aberrant ECM remodeling and hyaluronan production by fibroblasts, as well as tuft cell dysplasia, can further contribute to the disruption of alveolar function. Type II AEC-driven regeneration is a critical reparative response to infection-induced damage but can be overwhelmed in severe illness where there is extensive AEC cell death. IAV infection has also been found to compromise alveolar epithelium integrity in ways besides cell death, such as the direct disruption of cellular junctions in infected AECs. Loss of alveolar integrity and ensuing fluid leakage into alveolar luminal space are key features of viral pneumonia and ARDS. Overall, these changes to the lung epithelia highlight the intricate cascade of events and host–pathogen interactions that occur during IAV infection, particularly in cases of severe illness. Created with BioRender.com.

Cross-species transmission of IAV is typically non-productive, because of the lack of viral adaptation to the new host, but antigenic drift and shift can lead to the emergence of new strains with altered tropism and transmissibility. Antigenic drift is the result of accumulation of mutations in the viral genome over time and can be driven by evolutionary pressure to avoid host immunity, presenting a major cause of vaccine inefficacy.^16^ Antigenic shift can occur when a host is simultaneously infected by more than one strain of virus, enabling viral genome reassortment.^17^ This is thought to be a key mechanism in the emergence of pandemic strains of IAV, including the 2009 ‘Swine Flu’ pandemic strain which was a reassortant of avian, swine, and human viruses.^18^ Notably, incomplete adaptation to the human host may contribute to overactivation of the immune system and ensuing tissue damage characteristic of HPAI infections. Whether caused by excessive immune response or directly by IAV infection, lung tissue damage sustained during influenza (Fig. 1) underlies many of the clinical complications of severe illness in humans, including lung dysfunction, primary and secondary pneumonia, and acute respiratory distress syndrome (ARDS).^19,20^ These conditions often necessitate extended hospitalization and result in high rates of mortality in those with co-morbidities and other pre-disposing factors.^21^ This review will summarize what is currently known about IAV cell tropism in mammalian airway tissues, the fate of these cells following infection, and their roles in driving or mitigating influenza pathology.

## Airway epithelium

The conducting airway of the respiratory system includes the passages of the nasal cavity through to the branched airways within the lung. Lining these airways is an epithelium consisting of mostly columnar, pseudostratified cells. The conducting zone of the lung begins with the thick cartilage-supported tubing of the trachea which bifurcates into two bronchi, each leading into either the right or left lung. From there, the bronchi branch into a network of bronchioles, which finally end in the terminal bronchioles, supplying oxygen-replete air into the lung parenchyma. Besides simply lining the conducting airways, the airway epithelium provides a physical barrier protecting the lung and the rest of the body from particulate matter and pathogens, including influenza viruses.^22^ The major cells of the airway epithelium include ciliated, basal, goblet, club, tuft, and neuroendocrine cells. These cell types are present in varying proportions depending on the location within the airway tract.

### Ciliated cells

Ciliated cells are the most prevalent cell type of the airway epithelium, typically making up more than half of all cells found in these tissues.^23^ The cilia on the apical surface of these terminally differentiated cells function to provide the motive force needed to push the mucous coating of epithelium along the airway tract. Such mucociliary clearance (MCC) is the primary innate defense mechanism of the lung, helping clear pathogens (as well as abiotic contaminants) from the airways.^22^ Consequently, ciliated cells have an important role in initial defense against respiratory viruses, including IAV. Analyses of clinical samples, as well as findings from both in vivo and in vitro models, have established ciliated cells as targets of IAV infection.^24–26^ The susceptibility of ciliated cells to IAV infection varies, depending on the strain of the virus. In vitro studies have demonstrated that the relatively recent human H3N2 viruses are more efficient at infecting non-ciliated cells than human viruses from the 1968 pandemic, which showed a higher proclivity for ciliated cells.^25^ Recent in cellulo studies suggest ciliated cells may be a primary replicative niche for IAV, with a higher proportion of ciliated cells correlating with an increased viral burst size.^26^ Single-cell RNA sequencing (scRNA-seq) studies in mice have demonstrated that a small population of ciliated cells manifest detectable expression of interferon (IFN)-stimulated genes (ISGs) even in the absence of infection, suggesting these cells may play a role in the very early antiviral response.^24^ Following IAV infection, both directly infected and uninfected bystander ciliated cells upregulate ISG and inflammatory cytokine gene expression,^24^ indicating this cell type as a whole contributes significantly to antiviral response and immune cell recruitment. IAV infection of ciliated cells has been shown to interfere with ciliary function and likely impedes MCC.^27^ Recent work using recombinant IAV modified to encode a fluorescent reporter demonstrated that nearly 40% of ciliated cells were infected by 3 days post-infection (dpi) in a murine model.^24^ Although complete details of infected ciliated cell fate remain to be elucidated, it is likely that ciliated cells succumb to cell death upon infection. Support for this idea comes from the observation that IAV infection of lung epithelial tissue ex vivo results in significant loss of ciliated cells.^13^ Furthermore, activation of apoptosis via cleaved caspase-3 has been demonstrated in studies of IAV-infected porcine ciliated cells.^28^

### Goblet cells

Goblet cells and club cells are both secretory cells of the airway epithelium which, together, are estimated to constitute ~10% of epithelial cells by number based on scRNAseq analysis of healthy human tissue.^29^ The abundance of these two cell types varies depending on location within the airway tree. Goblet cells are the predominant secretory cell type in the larger airways, including in the trachea and bronchi. As the primary mucus-secreting cells of the lung, they cooperate with ciliated cells in the MCC function of the airway epithelium.^30^ A particular mucin produced by goblet cells, Mucin 5AC (MUC5AC), has been shown to be upregulated during IAV infections,^31^ and overexpression of Muc5AC was found to ameliorate influenza pathology in mouse models.^32^ While in vivo study of goblet cell fate during IAV infections is hampered by their paucity in the adult mouse pulmonary tissues, scRNAseq analyses have demonstrated that IAV infects goblet cells in cellulo.^12,26,33^ These studies have also shown that bystander uninfected goblet cell populations are capable of cytokine production in response to IAV infection.^33^ A significant decline in goblet cell numbers was also demonstrated post-infection,^33^ suggesting that this cell type may undergo significant cell death during IAV infection. However, direct in vivo evidence, or mechanistic details, of infected goblet cell death upon infection are lacking at present.

### Club cells

In the small airways of the lung, goblet cell numbers decrease, while club cell numbers increase. Club cells, formerly known as Clara cells, account for the majority of secretory cells present in the bronchiole-proximal epithelium.^34^ These cells produce essential factors important for lung function and protection, including the surfactant constituents of the airway epithelial lining fluid.^35^ Additionally, club cells express cytochrome P450 enzymes, which facilitate the detoxification of inhaled xenobiotics.^36^ Club cell infection by IAV has been demonstrated in vivo.^37,38^ Interestingly, certain club cell populations appear to survive this infection and manifest a robust type I IFN signature, expressing high levels of proinflammatory immunomodulators even long after virus has been cleared.^37^ Such post-viral inflammation has been suggested to represent a mechanism for protecting cells from reinfection during tissue repair.^38^ However, removing these ‘persister’ club cells after infection showed that these cells may increase inflammation and post-viral immunopathology.^37^ Pertinently, it has also been demonstrated that overactive IFN signaling may contribute to airway epithelium damage, independently of its immune-modulatory effects. Specifically, IFN anti-proliferative signaling may impede airway epithelial cell regeneration and consequently hinder lung airway tissue repair.^39^ Thus, club cells may be important in restricting the extent of IAV infection, but the proinflammatory persister cells that survive infection likely contribute to excessive inflammation and consequent tissue damage to the lung bronchi.^37,38^ More recent studies utilizing a Cre-inducible reporter mouse model indicate that several other cell types, including ciliated and AECs, may also elude lytic and CD8^+^ T cell-mediated clearance cell death to survive infection.^40^ It has been hypothesized that the general sparing of previously infected epithelial cells may decrease IAV-associated pathology.^40^ The extent of IAV-triggered cell death in club cells is currently unclear.

### Tuft cells

Tuft cells of the lung are a rare chemosensory cell type of the airway epithelium. They have an essential role in sensing allergens and inducing lung inflammation, and are thought to contribute to innate immunity within airways by modulating MCC.^41^ Recent studies utilizing murine lineage-tracing models and scRNAseq analyses, have begun to illuminate the fate of lung tuft cells during IAV infections.^42,43^ While pulmonary tuft cells are typically only found in the large airways, new populations of these cells arising within dysplastic regions of the distal epithelium have been documented in mouse models of severe influenza.^42^ These infection-induced tuft cells have been found in the alveoli of the lung, a region from which they are normally entirely absent.^43,44^ These expanded tuft cell populations may participate in promoting the lung pathology underlying ARDS, by enhancing plasma leakage into the alveolar air space.^42,43^ Currently, there is little data on whether tuft cells are infected by IAV, so it is unclear if direct infection of tuft cells plays a role in this dysplasia, or if it is primarily a response to tissue damage arising from infection and death of other cell types.

### Pulmonary neuroendocrine cells

Another relatively rare, yet important, sensory cell type in the airway epithelium are the pulmonary neuroendocrine cells (PNECs). Despite their paucity, PNECs are critical to lung function, as they are the only innervated cell type of the lung epithelium. PNECs sense and respond to both chemical and mechanical inputs, such as changes in O_2_ levels, or mechanical stretching of the airway epithelium.^45^ PNECs are thought to integrate such inputs and respond by secreting bioactive molecules including serotonin, γ-aminobutyric acid, and gastrin-releasing peptide.^46^ A recent study demonstrated that gastrin-releasing peptide secreted by PNECs during the course of infection may enhance lung pathology by increasing recruitment of inflammatory monocytes into pulmonary tissues.^47^ Likely in part due to their scarcity, little is currently known about the fate of PNECs during influenza, including whether they are directly infected by IAV or if they succumb to cell death upon infection.

### Basal cells

Basal cells are the resident stem cells of the lung epithelium. They make up nearly a third of the cellular composition in the larger airways, decreasing to ~6% towards the respiratory bronchioles.^48^ Basal cells have been found to act as progenitor cells for all major lung epithelium cell types. The efficient regeneration of the lung epithelia, enabled by basal cells, is critical to the preservation of epithelial barrier function in response to tissue damage from lung insults and diseases, including influenza.^28,49^ In particular, a subset of p63^+^ basal/basal-like cells have been shown to be critical in alveolar epithelium regeneration after severe IAV-induced damage.^49,50^ Club cells, descendants of basal cells, have also been identified as an additional stem cell population crucial to the regeneration of the lung epithelium after influenza illness and other lung diseases.^51,52^ Moreover, more recent studies indicate that it is primarily secretory cell-derived p63^+^ basal-like cells that contribute to alveolar repair.^53,54^ While the regenerative capacity of basal/basal-like cells is likely vital in the recovery from advanced flu illness, and in the prevention and recuperation from complications such as ARDS, there is evidence that this regeneration has the potential to become dysplastic and maladaptive, leading to fibrosis and persistent lung pathology.^55,56^ Though scRNAseq analysis of human lung epithelium has demonstrated that a small proportion of basal cells are indeed capable of becoming infected with IAV ex vivo, they appear to be relatively resistent.^26,33^ The extent of cell death induced in basal cells by IAV infection is currently unknown.

## Alveolar epithelium

Inhaled air is funneled through the upper respiratory tract and the conducting airways, after which it reaches the terminal bronchioles which outlet into the alveolar sacs of the lung. The alveolar sacs consist of central ducts branching into individual alveoli. The alveolar epithelium — the interior lining of alveoli — is the parenchyma of the lung wherein the core function of gas exchange takes place. This cellular lining is very thin to allow for the efficient exchange of gases, enabling the oxygenation of capillary blood.^57^ The primary cell type constituting most of the alveolar epithelium, by area, are the large, but diaphanous, type I AECs, which make up > 90% of the interior surface area of the alveolus.^58^ The other major cell type of the alveolar epithelium are type II AECs. Type II AECs are cuboidal in form and much smaller than type I AECs. While they only make up a small proportion of the alveolar epithelium by area, they account for ~60% of the epithelium by cell number.^59^

### Type I & II AECs

The unique morphology of Type I AECs allows these cells to form the honeycomb-like structure of the alveoli, which permit efficient gas flow from the alveolar lumen to the capillaries.^58^ Type II AECs have vital functions in the maintenance of the alveolar epithelium and the support of gas exchange by secreting various surfactants that coat the alveolar epithelium. These secreted proteins provide surface tension, prevent alveolar collapse, and are crucial in maintaining optimal gas exchange.^60^ Furthermore, secreted surfactants can mediate pathogen defense by trapping microbes and viruses and enabling their removal by immune cells.^61^ Type II AECs also transdifferentiate into type I AECs as a part of normal cell turnover and during alveolar regeneration following tissue damage.^62^

The alveolar epithelium warrants special focus on the study of influenza because alveolar damage and the resulting tissue dysfunction are arguably the primary determinants of severe disease.^63,64^ As the principal progenitor cells of the alveolar epithelium, type II AECs have a critical role in maintaining the epithelium and its function during influenza-induced lung injury by regenerating the tissue. In vivo studies have shown that proliferating populations of type II AECs are critical to alveolar repair during recovery from IAV-induced lung damage.^64,65^ Indeed, in a murine model of influenza, it was found that the primary causal factor behind the superior recovery of young vs aged mice, was the increased capacity of type II AECs to repopulate the alveolar epithelium.^66^

**Fig. 2:**
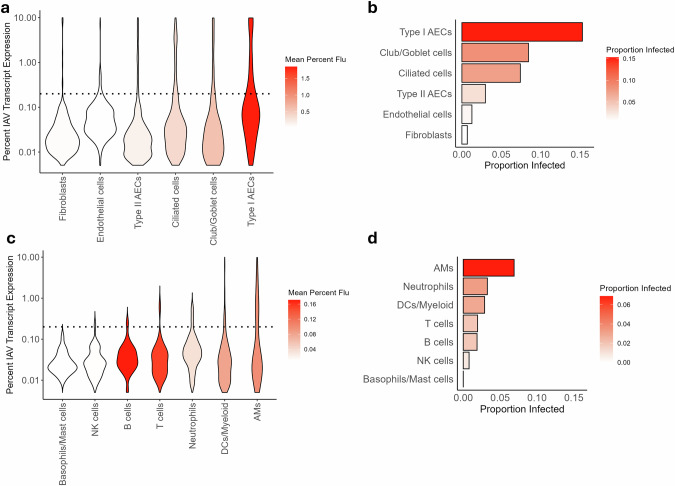
scRNAseq analysis of lung cells isolated from IAV-infected mice. **a**–**d** Adult (8–12-week-old) C57BL/6 mice were infected intranasally with a 2500 EID_50_ dose of mouse-adapted influenza A/Puerto Rico/8/1934 virus, and lung cells isolated from infected mice were profiled by scRNAseq analysis. Violin plots of percent total cellular transcripts mapped to IAV genes in lung structural (**a**) and immune compartment (**c**) cell types, plotted on log scale, range 0–10% (dotted line represents estimated infection threshold). Proportion of cells infected within individual lung structural (**b**) and immune compartment (**d**) cell types based on estimated infection threshold. Data are from combined 3 and 6 dpi datasets. Cells were isolated from the lungs of 4–5 mice for each time point. The data have been published previously^69^ (NCBI BioProject PRJNA613670).

While type II AECs have been proposed as dominant targets of IAV infection,^67^ recent scRNAseq studies have shown that both type I and II AECs are efficiently infected in murine infection models. In fact, type I AECs exhibited the highest degree of IAV positivity in these studies as determined by the detection of IAV transcripts in the scRNAseq data^68,69^ (Fig. 2). Viral antigen positivity has also been reported in both type I and II AEC cell types in patient tissue samples.^67,70^ While AEC cell death — whether programed or otherwise — is accepted to be a major cause of IAV-induced alveolar loss of function, it has also been demonstrated that viral infection may cause damage to the alveolar epithelium independently of cell death. Specifically, viral infection of AECs may directly impede the maintenance of claudin tight junctions, with data suggesting this in turn increases epithelium permeability.^71^

Diffuse alveolar damage and loss of AECs are characteristics of severe influenza disease and can ultimately lead to respiratory failure.^72–75^ In cases of mild damage caused by self-limiting infections, the proliferation and differentiation of local type II AECs into type I AECs is likely sufficient to restore tissue function. Alternately, in severe IAV infections, studies in murine models indicate that local type II AEC populations are largely ablated and instead lineage-negative progenitor epithelial cells are mobilized from the proximal regions of the respiratory tract to repair damaged alveolar tissue.^55,56^ This, however, appears to result in aberrant, and maladaptive, tissue repair.^55^ Additional studies provide evidence that the resulting areas of aberrant alveolar tissue, often characterized by fibrosis, persist long after the infection is cleared.^76,77^ In the most severe cases, alveolar damage may pass a point of no return. There is a clear relationship between extensive alveolar damage and patient mortality with significant AEC cell loss and death often evident in histopathological analyses of postmortem influenza patient samples.^73–75,78^ Furthermore, studies comparing murine models of mild vs severe IAV infection demonstrate major disparities in the loss of type I AECs, with a greatly increased loss of cells and decreased recovery evident in severe disease.^72^

While a complete understanding of the mechanisms that most contribute to AEC ablation has not yet been established, IAV infection has been shown to induce the activation of apoptosis in infected and uninfected AECs both in vitro, and in animal models.^79–81^ Infection of human type I AECs with IAV strains has been shown to induce intrinsic apoptosis through the BCL2-associated X protein (BAX)/Bcl-2 homologous antagonist killer (BAK)/caspase-9 cleavage pathway.^79,82^ Studies have implicated IAV proteins specifically in activation of cell-intrinsic apoptosis.^83^ Both PB1-F2 and NS1 IAV viral proteins have been found to mediate mitochondrial cytochrome c release.^84,85^ As to the observation of apoptosis induction in bystander (i.e., uninfected) AECs, this has been proposed to be explained, at least in part, by the activation of Fas and tumor necrosis factor (TNF)-related apoptosis-inducing ligand (TRAIL) receptors in lung epithelial cells as the result of ligand secretion from macrophage and other hematopoietic cell populations during infection.^81,83,86,87^

**Fig. 3:**
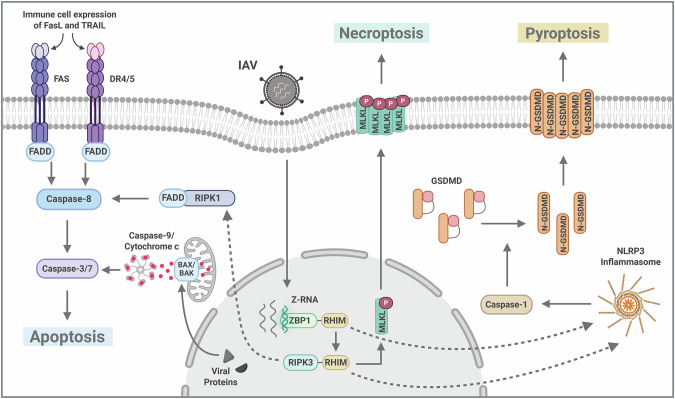
Programmed cell death pathways activated by IAV infection. IAV infection results in the production of Z-RNA, which is sensed by ZBP1. Once activated by binding Z-RNA, ZBP1 interacts with RIPK3 via RHIMs found in both proteins. RIPK3 can then induce either apoptosis via RIPK1-Fas-associated protein with death domain (FADD)-caspase-8 signaling, or necroptosis via mixed lineage kinase domain-like protein (MLKL). ZBP1-RIPK3 signaling can also activate the NLRP3 inflammasome to induce pyroptosis, via caspase-1 cleavage and activation of GSDMD. IAV infection can also induce intrinsic apoptosis via BAX/BAK/caspase-9 activation. Both PB1-F2 and NS1 proteins have been implicated in mediating mitochondrial cytochrome c release to trigger this pathway. Additionally, IAV infection can result in the initiation of extrinsic apoptosis via Fas ligand (FasL) and TRAIL-driven death receptor activation in lung cells. Created with BioRender.com.

In addition to apoptosis it has been shown, in recent studies utilizing murine models, that IAV infection can also readily induce necroptosis in type I AECs.^68^ These studies, and others, indicate that the specific mode of death induced in AECs, as well as other lung epithelial cell types, can significantly impact the severity of influenza illness.^68,88^ In particular, activation of inflammatory forms of cell death may provoke especially deleterious outcomes. Different forms of programmed cell death (PCD) vary widely in their immunogenic and inflammatory potential. For example, necroptosis and pyroptosis are both highly inflammatory forms of necrotic PCD, whereas apoptosis is considered relatively immunologically silent.^89^ The PCD pathways activated by IAV in relevant primary cell types (i.e., not cell lines) have begun to be elucidated, with the discovery that Z-form nucleic acid-binding protein 1 (ZBP1) and Receptor-Interacting Protein Kinase 3 (RIPK3) were essential for inducing most forms of IAV-triggered PCD^88,90^ (Fig. 3). IAV infection results in the production of Z-form RNA (Z-RNA) in the nuclei of infected cells.^91^ ZBP1 senses these Z-RNA species and binds RIPK3 via RIP homotypic interaction motifs (RHIMs). Once nucleated by ZBP1, RIPK3 becomes activated and induces either apoptosis or necroptosis.^92^ Additionally, ZBP1 activation has been shown to initiate NOD-, LRR- and pyrin domain-containing protein 3 (NLRP3)-driven pyroptosis in myeloid and airway epithelial cell lines.^88,90,93^ Although the exact mechanism by which ZBP1 activates the NLRP3 inflammasome is unclear, transforming growth factor-β-activated kinase 1 (TAK1) has been shown to suppress ZBP1-driven pyroptosis, as well as RIPK1/3-driven apoptosis and necroptosis in airway epithelial cell lines.^93,94^ Necroptosis activation in type I AECs and other cell types increases disease severity in mouse models of IAV infection.^68^ Compellingly, treatment of IAV-infected mice with a RIPK3 kinase inhibitor, which selectively blocks necroptosis, greatly blunted lung inflammation and pathology without affecting virus clearance or anti-IAV adaptive immune responses, and prevented lethality even when administered late in infection.^68^ Increased neutrophil infiltration, previously identified as a major driver of immunopathology in severe influenza,^95^ was significantly diminished in mice treated with the RIPK3 kinase inhibitor.^68^ Notably, pyroptosis also results in pathogenic neutrophil recruitment into pulmonary tissues.^96^ Preventing pyroptosis by ablation of gasdermin D (GSDMD) significantly reduced aberrant neutrophil recruitment and disease severity in IAV-infected mice.^96^ Altogether, the findings suggest that inhibitors of inflammatory PCD are potential therapeutics for severe influenza, either as monotherapies or combined with existing antivirals, such as ostelamivir (Tamiflu).

## Endothelium and connective tissue

### Endothelial cells

While epithelial cells have been established as the primary targets of productive influenza infection, other structural cell types of the lung have also been implicated in influenza pathology — even if the cells themselves are not permissive to IAV replication. This includes lung endothelial cells, which form the extensive capillary network surrounding the alveolar epithelium. Although capillary endothelial cells abut the epithelial replicative niche, only low levels of IAV RNA and IAV antigen have been typically reported in this cell type, suggesting that they are not especially permissive to IAV entry or that IAV infection is abortive.^69^ However, HPAI H5N1 viruses have been found capable of infecting the endothelium and infection of this cell type has been shown to be a major contributor to HPAI pathology. Additionally, endothelial cells are important sources of cytokine and chemokine (e.g., interleukin 6, C-X-C motif chemokine ligand 10) production during IAV infection.^97,98^ Furthermore, it was demonstrated that downregulating endothelial cell inflammatory signaling through Sphingosine 1-phosphate 1 receptor agonism significantly improved survival in mouse models of IAV infection.^98^ While in vitro studies have provided some evidence that IAV infection may induce apoptosis in endothelial cells, there is a lack of evidence that IAV infection induces significant cell death in endothelial cells in vivo.

### Fibroblasts and other mesenchyme-derived stromal cells

Pulmonary stromal cells associate closely with the endothelium and epithelium of the airway and alveolar interstitium. These mesenchyme-derived cells — which include alveolar fibroblasts, mesenchymal stromal (or stem) cells (MSCs), and pericytes — are the most abundant cell types of the interstitial space and are the primary producers of extracellular matrix (ECM). Excess production of the ECM component hyaluronan by fibroblasts (and other lung structural cells) during IAV infection may significantly contribute to pulmonary edema and ensuing pathology.^99^ In related findings, excessive degradation of the ECM by activated fibroblasts led to enhanced inflammation, independent of viral clearance in a murine model of IAV infection.^69^ In particular, the production of ECM protease A disintegrin and metalloproteinase with thrombospondin motifs 4 (ADAMTS4) by activated fibroblasts drove immunopathology and was predictive of disease severity in humans.^69^ In further mouse studies linking fibroblast subpopulations to IAV-induced pathology, Pdgfrb^+^ fibroblasts (derived from Pdgfra^+^ fibroblasts upon lung tissue damage), were shown to function with Krt5^+^ basal cells in driving dysplastic alveolar repair.^100^ These fibroblasts were found to highly express Notch receptors, resulting in a Notch-driven mesenchymal-basal cell niche wherein Wnt signaling (critical to functional epithelial regeneration) was significantly suppressed.^100^ While over-activation of certain fibroblast populations may drive excessive inflammation and dysplastic repair, fibroblasts may also have important functions in tissue regeneration post-infection. Studies of alveolar organoids (both mouse and human), indicate that fibroblast-secreted factors such as fibroblast growth factor 7 (FGF7) and FGF10 likely play a critical role in AEC repopulation.^65^ Further work, utilizing a murine model of IAV infection, revealed that besides pre-existing Fgf10-expressing alveolar fibroblasts, unique alveolar myofibroblast-like cells (AMFs) (derived from adult Gli1^+^ mesenchymal cells) were generated during infection.^101^ These AMFs may be a double-edged sword to IAV recovery as they were shown to support type II AEC growth ex vivo, but histological analysis of IAV ARDS patient lungs indicated that persistent activation of AMFs in severe infections may contribute to unresolved fibrosis and the development of ARDS.^101^ Although relatively limited, the existing research on MSCs in IAV infection suggests protective functions. In vitro studies indicate that MSCs may ameliorate IAV-infection impairment of alveolar fluid clearance.^102^ In another study using a pig model of IAV infection, administration of extracellular vesicles isolated from MSCs were found to inhibit virus replication and apoptosis induction in lung epithelial cells.^103^ This effect was apparently dependent on RNA transfer from extracellular vesicles to lung cells.^103^ Pericytes, another stromal cell of the lung, have a specialized role in modulating and supporting pulmonary blood vessels. Along with alveolar fibroblasts, pericytes have been shown to increase production of versican in response to type I IFN signaling in an IAV-infection mouse model.^104^ Versican is an ECM component with important functions in modulation of the immune response, with higher expression coincident with immune cell infiltration in lung disease.^105^ Pericytes are also producers of angiogenic factors during lung injury, including Angiopoietin-like 4 which has been shown to mediate alveolar tissue leakiness and damage.^106,107^ Although IAV induction of both apoptosis and necroptosis has been demonstrated in fibroblasts within in vitro contexts,^88,90,91^ it remains unclear if fibroblasts of the lung undergo PCD in significant numbers in clinical IAV infections. It is also currently unknown whether IAV efficiently infects or causes cell death in the additional stromal cell types of the lung.

## Resident and non-resident immune cells

### Dendritic cells

Lung-resident dendritic cells (DCs) are found throughout the lung, typically on basolateral side of the epithelium. These cells, along with alveolar macrophages (AMs), function as the major phagocytes of the lung under both homeostatic and disease conditions. Uptake and presentation of antigens from lung airways and alveoli by DCs enables tolerance to harmless antigens, while facilitating the activation of immune response to pathogens.^108^ During IAV infections, DCs are recruited to infected tissue by C-C chemokine receptor type 2, following which they become activated, take up viral antigen, and migrate to lymph nodes where they activate T cell-driven antiviral responses.^109^ In particular, CD11b^low/neg^CD103^+^ lung-resident DCs have been identified as a primary population of DCs that travel from the lung to the lymph nodes and present IAV antigen.^110^ Of note, DCs may contribute to Fas-FasL-mediated CD8^+^ T cell depletion during infection.^111^ Studies suggest that IAV infection of DCs may be largely abortive^112^ and, though it is difficult to disentangle phagocytosis from direct infection in phagocytic cells, studies in mouse models indicate that DCs can acquire IAV antigen through phagocytosis alone, without the need for direct infection.^113^ While likely not a major replicative niche, IAV has been shown to induce both apoptosis and necroptosis in mouse and human DCs in cellulo.^114^ Interestingly, it has been reported that pandemic strains of IAV suppress necroptosis in human DCs which may contribute to the pandemic potential of the virus.^114^

### Alveolar macrophages

AMs reside within the luminal space of lung alveoli in sparse numbers under homeostatic conditions, with approximately 1 AM per 3 alveoli.^108^ AMs have been found to have largely protective functions during influenza. Depletion of AMs in mouse models of IAV infection resulted in impaired viral clearance and increased rates of mortality.^115^ AMs are known to phagocytize infected apoptotic epithelial cells, prevent excessive neutrophil recruitment, and impede the development of pneumonia.^116,117^ In mouse models, AMs have been shown to be critical in limiting infection of Type I AECs and are thus vital in preventing severe pathology.^118^ Analysis of scRNAseq data shows that AMs are infected by IAV in the highest proportions amongst immune cell types in murine infection models (Fig. 2). There is also evidence from in cellulo studies that macrophages may function as productive replicative niches.^112^ Although AM levels drop precipitously early in infection, they are ultimately restored to homeostatic levels, in part as the result of infection-induced differentiation of monocytes into AMs.^116^ It has been demonstrated that IAV infection can induce PCD (apoptosis, necroptosis, and pyroptosis) in bone marrow-derived macrophages and human-derived AMs in cellulo.^90,119^ Additionally, macrophages appear contribute to pathogenic lung epithelial apoptosis by producing death receptor ligands, such as TRAIL and FasL.^81,83,86,87^

### Neutrophils

Neutrophils respond to viral infections in several ways, such as by degranulation, production of reactive oxygen species, formation of neutrophil extracellular traps (NETs), and phagocytosis of cell debris. Rapid and recurrent recruitment of large numbers of neutrophils is a major driver of IAV pathogenesis.^115^ While some studies suggest that neutrophils may limit IAV spread and minimize disease severity,^120^ others have shown that neutrophils drive influenza immunopathology and mortality.^68^ These opposing functions can be reconciled by findings indicating that while some neutrophil presence in the infected lung is beneficial, excessive and sustained recruitment of neutrophils is pathological. Indeed, studies of lethal influenza in mice have identified early and excessive neutrophil recruitment to lung interstitial tissue as a key determinant of severe disease.^95^ A cytokine- and pathogen-associated molecular pattern (PAMP)-driven feed-forward loop causing deleterious inflammation has been shown to underlie neutrophil-instigated pathology.^95^ In human peripheral blood samples, transcriptomic analyses revealed that expression of neutrophil-associated host factors, in particular, positively correlated with increased severity of IAV infection, while expression of genes associated with immune response overall actually declined with increasing disease severity.^121^ Furthermore, neutrophil dysfunction has been found to increase susceptibility to secondary bacterial pneumonia,^122^ where NETs may contribute to tissue damage.^117^ In studies comparing the effects of macrophage vs neutrophil depletion in IAV-infected mice, macrophage-depleted mice exhibited excessive neutrophil lung infiltration associated with increased alveolar tissue and capillary damage progressing to ARDS-like pathology.^117^ In these mice, extensive NET formation in the alveoli was observed. Alternately in neutrophil-depleted mice there was only mild infection-induced pathology.^117^ Comparisons of IAV-infection in aged vs young mice indicate that excessive neutrophil recruitment might play a role in increased mortality observed in elderly influenza patients.^123^ Increased and persistent neutrophil recruitment in aged mice was linked to increased numbers of senescent AEC cells which were found to secrete neutrophil-attracting chemokines, C-X-C motif chemokine ligand 1 and 2.^123^ Interestingly, neutrophil depletion after the onset of IAV infection significantly improved survival, whereas depletion prior to infection increased mortality.^123^ Further suggestive of the idea that neutrophils likely play a critical protective role during the early phase of infection, but that sustained or excessive neutrophil activity may significantly contribute to disease pathology. scRNAseq analysis of IAV-infected mice indicates that neutrophils have a relatively high infection susceptibility compared to other immune cell types (Fig. 2). Regarding infection-induced cell death, there are reports of IAV-induced neutrophil apoptosis.^120,124^ Furthermore, it has been shown that viral exposure increased neutrophil expression of Fas receptor and FasL, suggesting a potential mechanism for apoptosis induction.^124^

### Natural killer cells

Natural killer (NK) cells mediate anti-viral defense by targeting infected cells via antibody-dependent cellular cytotoxicity (ADCC) and ADCC-independent means. NK cells are estimated to comprise ~10% of all lymphocytes in the lungs of humans and mice under homeostatic conditions and are recruited into pulmonary tissues from peripheral circulation during inflammatory responses to lung insults, including IAV.^125^ The importance of NK cell ADCC in defense against IAV infection has also been demonstrated, with correlation between the presence of NK ADCC-promoting antibodies and reduced flu severity.^126^ Inhibitory and activating NK receptors mediate NK cell activation and cytotoxic activities. Mice lacking the activating receptor NKp46r are more susceptible to IAV-induced lethality than control animals.^127^ Besides these anti-viral activities, NK cells produce interleukin 22, which may promote epithelial cell regeneration and protection from pathological inflammation.^128^ Inversely, NK cell activity may also have deleterious effects on disease progression, particularly in more severe disease.^129^ NK cell depletion in a mouse model of IAV infection resulted in increased survival associated with reduced proinflammatory cytokine levels and decreased monocyte and neutrophil recruitment.^129^ IAV has been found to directly infect both murine and human NK cells.^130,131^ NK cell infection was shown to decrease NK cell cytokine production and cytotoxicity, as well as trigger NK cell apoptosis, although the specific mechanism(s) of apoptosis induction remain to be identified.^130,131^

### T cells

Adaptive response to IAV infection begins with T cell recruitment, which starts as early as 3 dpi, and peaks several days later.^132^ CD8^+^ T cell recruitment has been found to correlate with decreased disease severity and improved recovery.^133^ In murine models, CD8^+^ cytotoxic T lymphocytes were shown to be protective at low viral dose but contributed to influenza-induced pathology at high viral dose.^134^ In particular, influenza-specific, polyfunctional T cell recruitment appears to be protective, while the activation of non-antigen-specific T cells and other non-specific immune responses are associated with increased IAV infection susceptibility.^135^ Memory CD8^+^ T cells have been associated with critical protection against IAV infection,^136^ and diminished levels of this cell type with age has been proposed to mediate decreased IAV antiviral immune response in older patients.^137^ In particular, tissue resident memory T cells (T_RM_) appear to be vital in limiting infection. Studies of murine models of IAV infection indicate that T_RM_ cells mount a robust response, and while significant numbers of systemic memory T cells are recruited to the lung, they may be largely dispensable to viral clearance.^138–140^ Furthermore, studies comparing mice immunized via intraperitoneal vs intranasal routes demonstrate that T_RM_ are not only critical in restricting viral replication but also in mediating protection to subsequent IAV infection, indicating that the ability to generate IAV-specific T_RM_ may be key to efficacious anti-IAV vaccines.^141^ Notably, rather than direct cytotoxic activity, production of IFN-γ, and the subsequent activation of an antiviral state in surrounding epithelial cells, has been found to be the primary mechanism of T_RM_-mediated protection.^141^ Regulatory T cells have been found to be vital in the generation of memory T cells following influenza.^142^ Additionally, sustained expression of interferon-induced transmembrane protein 3 was found to be important in maintaining anti-influenza CD8^+^ T_RM_ cells by protecting against infection in these cells themselves.^143^ Notably, CD4^+^ T cell function in anti-IAV immunity is not limited to helper T cell activity, as CD4^+^ T cells were found to be protective against IAV pathology independent of CD8^+^ T cells.^144^ Furthermore, preexisting CD4^+^ T cells, but not CD8^+^ T cells, were found to be critical to viral clearance and limiting disease severity in clinical studies.^145^ Apart from these protective functions, studies have also implicated T cells in IAV-induced immunopathology. In a study of lung tissue from patients with influenza-associated lung fibrosis, a correlation was found between excessive CD8^+^ T cell infiltration and, both, fibrosis and aberrant immune-epithelial progenitor niches.^146^ Furthermore, using a mouse model of post-viral fibrosis, exuberant CD8^+^ T cell activity was shown to impair recovery through a CD8^+^ T cell-macrophage-epithelial progenitor axis.^146^ Specifically, CD8^+^ T cell production of IFN-γ and TNF was found to promote macrophage expression of interleukin-1β, which in turn was shown to be a negative regulator of type II to type I AEC transdifferentiation.^146^ In a study that compared IAV-infection of aged vs young mice, transforming growth factor-β receptor signaling was found to drive an increased accumulation of T_RM_ in aged mice, which in turn resulted in higher levels of lung inflammation and fibrosis.^147^ It was additionally found that, unlike the CD8^+^ T_RM_ in young mice, IAV-specific CD8^+^ T_RM_ of aged mice were not protective against rechallenge — apparently due to a lack of effector capacity.^147^ In cellulo studies of peripheral blood mononuclear cells (PBMCs) have demonstrated T cell-specific infection by IAV, and shown that viral exposure activated T cell apoptosis, potentially induced by monocyte-macrophage surface expression of viral neuraminidase protein.^148^ Transient leukopenia is known to be characteristic of human influenza disease and significant lymphocyte depletion by apoptosis has been observed in mouse models of IAV infection.^149^ In vitro studies utilizing human PBMCs indicate that IAV exposure can induce significant levels of apoptosis in bystander (uninfected) T cells, mediated largely by Fas.^149^ Indeed, FasL-expressing DCs were shown to increase mortality in IAV infection mouse models via the elimination of virus-specific CD8^+^ T cells.^111^ Furthermore, studies in aged mice indicate that increased susceptibility of CD8^+^ T cells to apoptosis may contribute to worse influenza outcomes with age.^150^

### B cells

Effector B cell production of virus-specific antibodies provides protection from initial infection, aids in the control of viral spread, and contributes to ultimate infection resolution. In the case of IAV, virus-specific antibodies are often generated against viral hemagglutinin proteins.^151^ The generation of IAV-specific B cells is thought to be a key factor in influenza vaccine protection and impaired memory B cell function has been linked to decreased flu vaccine response in the elderly.^152^ While IAV-induced lung T_RM_ have long been established, studies in recent years have also provided clear evidence of lung-resident memory B cells (B_RM_).^153–156^ Analogous to T_RM_, studies in murine models indicate that local IAV antigen exposure within the lung is required for B_RM_ generation.^153,156^ Evidence also suggests that B_RM_ reside permanently in lung and are not recirculated.^153^ Use of Aicda-reporter mice has shown that IAV infection induces both IAV-specific B_RM_ and bystander B_RM_ (which have no specificity for IAV) and that, upon reinfection, IAV-specific B_RM_ (particularly IgG isotypes) can readily differentiate into plasma cells providing rapid production of protective antibodies.^154^ Further studies in mice have demonstrated that B_RM_ are able to migrate to alveoli via IFN-γ and C-X-C motif chemokine receptor 3 signaling from AMs, locally differentiating into plasma cells at specific sites of infection/viral replication.^155^ Single cell analyses of both mouse and human B cells indicate the presence of transcriptionally distinct lung B_RM_ after IAV infection, with conserved expression of CD69 specific to lung B_RM_ (as well as lung draining lymph node memory B cells) across both species.^156^ Studies in murine models indicate that B cells also play a role in regulating lung macrophage response early in IAV infection. Specifically, acetylcholine produced by B cells was found to dampen macrophage production of TNF — which if unchecked — hampered epithelium repair, indicating an important role for B cells in limiting pathological inflammation.^157^ There is currently limited data available as to whether B lymphocytes are significantly infected by IAV, apart from in vitro studies demonstrating monocyte-macrophage dependent lymphocyte infection in isolated PBMCs.^158^ As with T cells, B cell populations in isolated PBMCs have also been shown to undergo Fas-mediated apoptosis upon exposure to IAV.^149^ Additionally in murine models, IAV infection was found to significantly deplete bone marrow pre/immature B cell populations via tumor necrosis factor receptor-dependent apoptosis.^159^ Interestingly both B and T cells have notably high levels of constitutive ZBP1 expression,^160^ but whether either cell type undergoes necroptosis during IAV infection remains to be seen.

## Conclusions

Mouse models enabling lineage tracing and manipulation of specific cell populations, as well as advances in single-cell technologies, have greatly enhanced our understanding of the course of influenza infection and the ensuing host response at a cellular level. While most immune cells, fibroblasts, and endothelial cells are not thought to be efficiently infected or productive replicative niches, almost all the major lung epithelial cell types have been found to be susceptible to IAV infection. However, the fates of these cells after infection appear to be highly varied. In the case of AECs, influenza infection can induce significant cell loss, particularly in the case of severe disease. This damage to the alveolar epithelium can lead to the loss of lung function and is thought to underly major influenza complications including pneumonia and ARDS. Interestingly, infection does not necessarily result in cell death in some epithelial cell types, such as club, ciliated, and alveolar epithelial cells. Furthermore, cytokine signaling from these infection ‘survivor’ cells can go on to mediate immune cell activation and even drive immunopathology. Additionally, findings suggest that cells such as the ciliated cells of the lung demonstrate significantly varied infection susceptibility and, thus, fate depending on the infecting IAV strain.

The variability in human cell susceptibility and response complexity to IAV infection may reflect the fact that humans are relatively recent hosts for IAV. Wild aquatic birds are considered the major ecological niche of IAV, wherein IAV replicates primarily in the gastrointestinal tract.^161^ IAV strains spilling over from the avian niche thus face a massively altered cellular environment when they infect mammalian lungs. A lack of complete host adaptation may be a significant factor in the severe immunopathology exhibited in highly virulent pandemic strains of recent zoonotic origin. This enhanced pathology underlies the increased incidence of serious complications and mortality during pandemics.

Overall, the studies reviewed here have identified compelling new potential avenues for therapeutic targeting of host inflammatory responses, rather than virus itself. Several of the therapeutic strategies suggested by these studies — such as inhibition of inflammatory PCD,^68^ cytokine signaling,^98^ and ECM modification^69^ — have shown significant promise in experimental models and act by ultimately limiting excessive immune cell activation, thereby mitigating immunopathology induced by influenza. Other findings, such as impaired epithelial regeneration due to IFN anti-proliferative signaling,^39^ indicate that targeting the host antiviral response, independently of immune cell modulation, may also have significant benefit.
